# Emotional Nuance: Examining Positive Emotional Granularity and Well-Being

**DOI:** 10.3389/fpsyg.2022.715966

**Published:** 2022-02-22

**Authors:** Tse Yen Tan, Louise Wachsmuth, Michele M. Tugade

**Affiliations:** Vassar College, Poughkeepsie, NY, United States

**Keywords:** emotion, positive emotion, granularity, well-being, emotion differentiation

## Abstract

The focus of this review is on positive emotional granularity. Emotional granularity is the level of specificity that characterizes verbal representations of an affective experience. Although there has been research on negative emotional granularity, relatively less attention has been given to the study of positive emotional granularity. Positive emotions are theorized to motivate an individual to “broaden and build” one’s scope of cognition, attention, and behavior. Distinct positive emotion concepts may provide individuals with more informational value than that provided by global mood. Indeed, individuals who are higher in positive emotional granularity report being better at coping with stressful experiences. In this review, we discuss growing research on positive emotional granularity and well-being. Issues of measurement, interventions, and considerations for future lines of research are discussed.

## Introduction

The global pandemic has been emotional. Consider different emotional reactions in response to health and safety ordinances. One response might reflect more broad categories of feeling: “I don’t want my stress level to be so high. I want this nightmare to be over.” Another response might reflect more nuanced feelings: “I feel sad or I feel anxious and I don’t like that feeling, so I get angry at the person (for not wearing a mask)” (Powell, 2020). Lay people, writers, poets, and scientists alike tend to agree that words like happiness, interest, gratitude, anger, sadness, and fear represent distinct kinds of experiences. Yet, people vary in the degree to which they use emotion words to represent separate, discrete experiences (Feldman, 1995; Barrett, 1998, 2004), as seen in the various responses above. This phenomenon is called “emotional granularity” (Barrett, 2004).

Emotional granularity is the level of specificity that characterizes verbal representations of an affective experience. When asked to report how they feel, some individuals use emotion words like “happy,” “excited,” “sad,” and “angry” to represent highly differentiated experiences. These individuals are higher in emotional granularity, and report their emotional experience in more precise, differentiated terms, using discrete emotion labels like happy, sad, angry, etc., in a way that captures the distinctiveness in these words. Others represent their experiences in more global terms. These individuals are low in emotional granularity: they reported their experience in global terms, using discrete emotion labels to communicate only the most general of information (typically, pleasure and displeasure). They use words like “angry,” “afraid,” and “sad” interchangeably, not distinguishing between discrete emotion terms.

Recent research examining the neural mechanisms underlying granularity shows that emotional granularity extends beyond exclusively verbal representations (Lee et al., 2017). Lowly and highly granular individuals show different patterns of neural activity as their brains represent emotional experiences. When presented with emotional stimuli, highly (vs. lowly) granular individuals evidenced sustained attention and executive control to access conceptual knowledge to make meaning of affective stimuli. As such, the mechanisms of emotional granularity can be captured by neural processing beyond the labeling of emotions, *per se*. These findings show that emotional granularity is the tendency to experience emotion in a highly specific manner (e.g., Lee, et al., 2017).

Emotional granularity has been increasingly associated with social and emotional well- being, and has been theorized to be a key aspect of emotion regulation (Kalokerinos et al., 2019). For instance, individuals high in granularity have been found to possess greater emotion regulation skills (Barett et al., 2001), while low granularity is associated with poor emotion regulation strategy effectiveness (Kalokerinos et al., 2019). The role emotional granularity plays in emotion regulation may be attributed to the feelings-as-information theory(Schwarz, 2012), in that differentiation provides individuals with a better understanding of the cause of the emotion, and therefore facilitates contextually sensitive emotion regulation.

Emotional granularity can refer to the differentiation of both positive and negative emotions. Negative emotional experiences prompt individuals to narrow specific thought-action repertoires, conferring immediate adaptive effects on the individual; however, feeling angry should lead to a vastly different response than feeling afraid (e.g., fight vs. flight). The focus of this review is on positive emotional granularity. Positive emotions are theorized to motivate an individual to “broaden and build” one’s thinking, attention, and behavioral repertoires (Fredrickson, 2001; Tugade et al., 2004). Much like in the case of negative emotions, discrete positive emotion concepts may provide individuals with more useful information than that provided by global mood. For instance, individuals who are higher in positive emotional granularity report being better at coping with stressors (Tugade et al., 2004).

## Benefits of Positive Emotional Granularity

Growing research in the field suggests that individual differences in emotional granularity are associated with emotion regulation and psychosocial adjustment. Individuals may differ in how emotionally granular they are. One theory cited in the literature that allows for the exploration of these differences is the Theory of Constructed Emotion, as elucidated by Barrett (2006), wherein emotional concepts are cognitively and socially constructed. Barrett (2006) notes that differences in experiences of emotion can reflect individual differences in perceived intensity and frequency of felt affect, with some individuals reporting their experiences on positive vs. negative dimensions (e.g., pleasant vs. unpleasant), while others report more nuanced, granular experiences (e.g., content, joyful, sad, angry). Studies suggest that emotional granularity may facilitate adaptive coping and successful self-regulation (Kashdan et al., 2015). Research into emotional granularity is still limited, and much of the research has focused on negative emotional granularity. However, emerging evidence shows that positive emotional granularity- though less studied - can be beneficial in its own right.

While emerging theories discuss individual differences in positive emotional granularity, other theoretical works focus on the distinct functions of discrete positive emotions. The theories described below focus on the importance of understanding the unique function of discrete positive experiences. A compelling argument for examining positive emotion differentiation can be found in Ellsworth and Smith (1988) study on positive emotions and associated patterns of appraisal. Though positive emotional experiences were found to be somewhat less differentiated than negative emotional experiences, considerable differentiation was still present, and different positive emotions were found to have distinct patterns of appraisal consistent with the emotions’ proposed adaptive functions (Ellsworth and Smith, 1988). Furthermore, Shiota et al. (2014) theorize that there are distinct adaptive functions of discrete positive emotions. In their PANACEAS taxonomy (an acronym that represents eight different positive emotions: pride, amusement, nurturant love, attachment love, contentment, enthusiasm, awe, and sexual desire), Shiota et al. (2014) explore the events that the aforementioned positive emotions might be responsed to, and then posit potential adaptive responses to that event. For instance, awe is experienced when one encounters novel and complex information about the world beyond one’s previous knowledge and understanding; an adaptive response to awe might be to form new schemas about the world (Shiota et al., 2014). Yet another approach is Fredrickson’s Broaden-and-Build theory, which posits that positive affective experiences broaden people’s momentary thought-action repertoires and serves to help individuals build personal resources (Fredrickson, 2003). For instance, Fredrickson (2003) notes the thought-action tendency and the personal resources accrued for pride to be different from joy. Taken together, the work by Ellsworth and Smith (1988), Shiota et al. (2014), and Fredrickson (2013) all point to how the appraisal, adaptive functions, and experience of different positive emotions can lead to different outcomes. Positive emotion differentiation therefore may play an important role in long-term thriving, as specific positive emotions may signal potential context-dependent benefits and can influence one’s behavioral intentions (Soscia, 2007).

Positive emotional granularity may thus have benefits in terms of social relationships. Aptitude in emotional granularity may translate to one having a more accurate understanding of the emotional states of others, and facilitate interpersonal communication; studies have found that individuals high in emotion differentiation were more able to accurately categorize and recognize others’ facial expressions, and individuals with high emotional granularity were better able to judge the emotions of their romantic partners (Erbas et al., 2016; Israelashvili et al., 2019). Though these studies focused on negative emotion differentiation, differentiating between one’s positive emotions may similarly be beneficial to understanding the emotions of others.

According to the emotions-as-information theory (Schwarz, 2012) a heightened ability to differentiate between positive emotions may lead to a range of different affective and cognitive responses depending on the specific emotion. Algoe et al. (2010) found that experiencing feelings of gratitude in the context of a romantic relationship has been associated with increased feelings of relationship quality. Research shows that in individuals with anorexia, those with low positive emotional differentiation engaged in more vomiting, laxative use, exercising, weighing, restricting, and checking for fat. Furthermore, individuals who reported higher positive emotion intensity and low positive emotion differentiation engaged in even more frequent maladaptive weight-loss behaviors, suggesting that the effect of positive emotion intensity on weight-loss behaviors is moderated by low positive emotion differentiation. Selby et al. (2014) theorize that the unhealthy behaviors are due to the individuals’ attribution of general positive mood about reaching their weight-loss goals to specific positive emotions such as confidence, accomplishment, and happiness. This misattribution and over association of a broad range of positive emotions with the achievement of a weight-loss goal therefore leads to the perpetuation of unhealthy behaviors. Because of the unique functions and informational value of specific positive emotions, high positive emotional granularity is especially important as the lack thereof may limit or misguide one’s behavioral responses in a given situation.

Additionally, Bao and Lyubomirsky (2013) have posited that increasing the number of positive events and emotions in a relationship may combat hedonic adaptation, leading to sustained relationship well-being. One could hypothesize that experiencing a diversity of positive emotions would also be useful in building resistance to one’s adaptation to a positive emotional experience, building on previous findings that suggest that novelty can reduce habituation (Leventhal et al., 2007). Positive emotional differentiation may also lead to better health outcomes: Quoidbach et al. (2014) found that positive emodiversity was negatively related to annual visits to family doctors, days spent in the hospital per year, and the mean defined daily dose of medication, in contrast to mean positive emotion, which was not significantly related to any of these indicators of health. While emodiversity and emotional granularity are different concepts, the constructs seem to be linked. To experience a diverse range, and abundance of emotions, or to have high emodiversity, likely requires that an individual be able to differentiate between varied emotions, rather than experiencing general, global moods. See Table 1 for a summarized list of these hypothesized benefits of positive emotional granularity alongside their theoretical approaches.

**TABLE 1:** Hypothesized benefits of positive emotional granularity and supporting theoretical approaches.

Possible benefits of positive emotional granularity	Approaches/Theories
Social connections, e.g., improved relationship quality	Broaden-and-build theory (Fredrickson, 2004)
	Emotions-as-information theory (Schwarz, 2012)
Combat hedonic adaptation	Hedonic adaptation prevention model (Lyubomirsky, 2011; Bao and Lyubomirsky, 2013)
Physical health	Emodiversity (Quoidbach et al., 2014)
	Emotions-as-information theory (Schwarz, 2012)

## Positive Emotional Granularity Across Cultures

Another relevant area of positive emotional granularity research is cross-cultural research, which can reflect different nuances in positive emotional experiences. Given the benefits of emotional granularity, it is likely important and present across cultures; however, it is important to consider cross-cultural factors that may reflect differences in emotional experiences, such as differences in the perceived affective valence (An et al., 2017), and physiological arousal in response to a given experience (Lim, 2016). For instance, An et al. (2017) found cross-cultural differences in the perceived degrees of “positivity” and “negativity” of six “basic” emotions (sadness, fear, disgust, anger, surprise, and happiness) Ekman, 1992. While basic emotions may be common globally, the interpretation and perception of said experience can differ: Chinese individuals view happiness as a harmonious, homeostatic state, and understand that pursuing happiness may not always be positive. In contrast, Americans describe happiness as more emotionally charged (Lu and Gilmour, 2004), and pursuing happiness is considered desirable. Drawing on the constructionist theory of emotion (Barrett, 2006; Lindquist and Barrett, 2008), language, and concepts (that may be socially learned) constitute the experience and perception of emotion, by helping one to make meaning of internal sensations and external stimuli. Taken together, the research on cultural differences in positive emotional experience has important implications for the study of positive emotional granularity. One’s propensity for emotional granularity, may vary because of differences in attention to different facets of an emotional experience, which may be influenced by philosophical traditions (e.g., Confucianism, Buddhism; Lu and Gilmour, 2004; Zhou et al., 2021), education, and language. In addition, differences in emotional experiences may also have implications for subsequent behavioral responses and emotion regulation.

Positive emotional granularity might also be reflected *via* non-verbal channels. There are different ways that people might express feelings of joy, surprise, and amusement that are context-dependent, therefore differentiation of positive emotions (both in oneself and in others) must be culturally informed. For instance, Jack et al. (2012) found differences in facial expressions between individuals from Western and Eastern cultures. Eastern individuals were found to express emotional intensity primarily with eye activity (Jack et al., 2012); these results corroborate previous studies exploring cultural differences in recognizing facial expressions, where Eastern groups were found to fixate on the eye region (Jack et al., 2009). Similar to facial expressions, non-verbal emotional vocalizations are a means of communicating specific affective states. In a study conducted by Sauter et al. (2010), while “basic emotions” were recognized *between* cultural groups, vocalizations of positive emotions were specific *within* cultural groups. Noting the variability in expression and perception is important, especially when communicating differential positive emotions (Sauter, 2017). Together, these studies indicate that the study of positive emotional granularity in cultures is important because it can demonstrate that granularity is expressed and communicated differentially between individuals within a cultural group (e.g., upper face expressions, vocalizations).

## Measuring Emotional Granularity

Many studies have used experiential sampling methodology to measure positive emotional granularity. Experience sampling methodology (ESM) gathers data on individuals’ feelings, thoughts, and actions in the context of everyday life. Applying ESM to positive emotional granularity allows researchers to measure the differentiation of emotions as they are experienced, *in situ*, in the context of daily life. Participants rate their momentary emotional experience multiple times a day, for several weeks. Using ESM, researchers may compute an emotion differentiation index based on the interrelatedness of similarly-valenced emotion terms (Barett et al., 2001; Tugade et al., 2004). Greater interrelatedness when reporting positive emotional words would reflect lower emotional granularity (less differentiation); whereas lower interrelatedness when reporting positive emotional experience reflects higher emotional granularity (more differentiation). Such methods capture momentary emotion differentiation, in an ecologically valid context, and also makes use of new technologies at hand.

Previous measures of granularity have relied mainly on experience-sampling methods (e.g., Barett et al., 2001; Tugade et al., 2004). ESM offers an important advantage in that the measures are based on participants’ actual emotional experiences as they unfold over time, revealing unique patterns of emotional experience within each individual. Even still, this approach has some limitations. First, because the experiences sampled are those that arise spontaneously across different contexts in respondents’ lives, the set of experiences can vary greatly from individual to individual, sometimes rendering the scores difficult to compare across individuals. There would be considerable utility in differentiation scores derived from a common set of experiences. Second, ESM can be time-consuming, expensive, and difficult to implement. Thus, ESM can limit the range of studies to measure positive emotion granularity and associated factors.

As research on positive emotion granularity increases, researchers have developed new measures of positive emotional differentiation. One such measure is the Differentiation of Positive Emotion Scale (DOPES), wherein individuals are asked to imagine and indicate their emotional reactions to a set of eight positive emotion-eliciting vignettes (Kirby et al., 2014). Each vignette is designed to elicit a specific positive emotion, and respondents are asked to rate their emotional reaction based on eight targeted emotions: happiness, pride, gratitude, interest, hope, challenge/determination, awe, or contentment. This self-report measure can be used to assess individual differences in positive emotional granularity. For each vignette, respondents are asked to rate their imagined emotional responses in terms of each of the eight targeted emotions. The degree of emotion differentiation (granularity) for each respondent is quantified by intercorrelating the ratings for each emotion scale across the eight vignettes, then computing the mean intercorrelation. To normalize the distribution of the resulting scores, this average correlation is subjected to an *r*-to-*z* transformation. Higher mean intercorrelations reflect *lower* levels of differentiation because they indicate that the emotion ratings covary strongly across the vignettes (Kirby et al., 2014). Thus, in addition to ESM, the DOPES is another viable measure of the tendency to differentiate positive experiences.

## Positive Emotional Granularity Interventions

Although research on the benefits of emotional granularity is growing, there has been little focus on ways to improve emotion differentiation ability, and the research on positive emotional granularity interventions is especially sparse. Mindfulness techniques may be useful in contributing to more positive emotional differentiation. In an empirical study of a mindfulness- based intervention, Van der Gucht et al. (2019) hypothesized that mindfulness may contribute to better positive and negative emotion differentiation. Significant improvement in both positive and negative emotion differentiation was found, although the study did not include a control group. Improvements in negative emotion differentiation were found both post intervention and at a 4 month follow up, although after controlling for negative affect levels this improvement was no longer significant. Improvement in positive emotion differentiation was only significant at the 4 month follow up, and was significant even after controlling for mean positive affect levels. Van der Gucht et al. (2019) speculate that these findings may mean that positive emotion differentiation takes more time to learn as compared to negative emotion differentiation, or that there is more opportunity to improve negative emotion differentiation. While the results of this study are complex and findings may differ when including a control group, the findings indicate that mindfulness-based interventions may be useful tools in improving emotion differentiation (Van der Gucht et al., 2019). Programs focused on emotional intelligence may also be relevant to emotional granularity and often include aspects of emotion differentiation training.

Programs that promote emotional intelligence and social emotional learning often also focus on emotion labeling and differentiation. While many of these interventions do not directly measure emotional granularity, they may be useful to improve emotional granularity and often focus indirectly on emotion differentiation. Emotional intelligence training has been shown to lead to improvement in emotion identification and differentiation (Nelis et al., 2009). RULER is one such intervention that may improve emotional intelligence as well as emotional granularity. For example, the RULER feeling words curriculum focuses on teaching students about feeling or emotion words, what they mean, and how to label their emotions accurately. Each unit includes multiple different lessons and activities integrated into classroom instruction focusing on specific feeling or emotion words (Brackett et al., 2012). Integrating these types of curricula may help students correctly label and differentiate between their emotions. Broadening children’s understanding and use of different emotion words and correctly labeling their emotions using the RULER feeling words curriculum has led to improved academic performance and social behavior (Brackett et al., 2012).

Interventions targeting emotional differentiation may be useful for adults in the workplace. In fact, one study found that employees who participated in an emotional intelligence intervention which included a focus on emotion differentiation had increased work performance scores after participating in the intervention (Munir and Azam, 2017). Interventions that include a focus on emotional granularity should be developed and implemented across the lifespan. A focus on positive emotion differentiation especially may have benefits in terms of social relationships. Interventions that ask individuals to differentiate between positive emotions and to reflect on the functionality of their felt emotional experiences (Shiota et al., 2014) may also be especially valuable. This approach may help individuals learn about why certain distinct positive emotions (e.g., pride vs. gratitude) can be adaptive in various contexts of one’s daily life.

## Conclusion and Future Directions

The present paper sets the stage for future research directions. For instance, it would be fruitful to disentangle the effects of state versus trait positive emotional granularity. It may be possible that particular contexts allow one to be more emotionally granular than others, and this might have implications for emotion regulation. Second, it would be important for future work to experimentally manipulate positive emotional granularity. Experimental manipulations of emotional granularity would allow future research to make causal claims about the consequences of emotion differentiation. Indeed, because emotional granularity has been studied as an individual difference construct as measured in the context of daily life, such manipulations would be complex and require further investigation. Third, it would be important to investigate the processes that allow for positive emotional granularity. We speculate that emotional granularity may reflect individual differences in complex emotion knowledge or cognitive resources that are useful in appropriately and effectively navigating through one’s daily life. Empirical work on this theory could reveal important psychological and cognitive processes that link positive emotional granularity with emotion regulation. Fourth, it would be important to examine the developmental trajectory of emotional granularity. There may be individual differences in people’s capacities to be granular for positive versus negative emotional experiences. Understanding mechanisms associated with such differences would elucidate the developmental path that enables one to achieve high levels of precision in their representations of emotion.

## Author Contributions

MT, TT, and LW contributed to the conception and design of the manuscript and wrote sections of the manuscript. All authors contributed to manuscript revision, read, and approved the submitted version.

## Conflict of Interest

The authors declare that the research was conducted in the absence of any commercial or financial relationships that could be construed as a potential conflict of interest.

## Publisher’s Note

All claims expressed in this article are solely those of the authors and do not necessarily represent those of their affiliated organizations, or those of the publisher, the editors and the reviewers. Any product that may be evaluated in this article, or claim that may be made by its manufacturer, is not guaranteed or endorsed by the publisher.

## References

[B1] Algoe S. B. Gable S. L. Maisel N. C. (2010). It’s the little things: Everyday gratitude as a booster shot for romantic relationships. *Pers. Relationsh.* 17 217–233.

[B2] An S. Ju L. Marks M. Zhang Z. (2017). Two sides of emotion: exploring positivity and negativity in six basic emotions across cultures. *Front. Psychol.* 2017:610. 10.3389/fpsyg.2017.00610 28473791 PMC5397534

[B3] Bao K. J. Lyubomirsky S. (2013). Making it last: combating hedonic adaptation in romantic relationships. *J. Posit. Psychol.* 8 196–206. 10.1080/17439760.2013.777765

[B4] Barett L. F. Gross J. Christensen T. C. Benvenuto M. (2001). Knowing what you’re feeling and knowing what to do about it: mapping the relation between emotion differentiation and emotion regulation. *Cogn. Emot.* 15 713–724.

[B5] Barrett L. F. (1998). Discrete emotions or dimensions? The role of valence focus and arousal focus. *Cogn. Emot.* 12 579–599.

[B6] Barrett L. F. (2004). Feelings or words? Understanding the content in self-report ratings of experienced emotion. *J. Personal. Soc. Psychol.* 87:266. 10.1037/0022-3514.87.2.266 15301632 PMC1351136

[B7] Barrett L. F. (2006). Solving the emotion paradox: categorization and the experience of emotion. *Personal. Soc. Psychol. Rev.* 10 20–46. 10.1207/s15327957pspr1001_2 16430327

[B8] Brackett M. A. Rivers S. E. Reyes M. R. Salovey P. (2012). Enhancing academic performance and social and emotional competence with the RULER feeling words curriculum. *Learn. Indiv. Diff.* 22 218–224. 10.1016/j.lindif.2010.10.002

[B9] Ekman P. (1992). An argument for basic emotions. *Cogn. Emot.* 6 169–200.

[B10] Ellsworth P. C. Smith C. A. (1988). Shades of joy: patterns of appraisal differentiating pleasant emotions. *Cogn. Emot.* 2 301–331. 10.1080/02699938808412702

[B11] Erbas Y. Sels L. Ceulemans E. Kuppens P. (2016). Feeling me, feeling you: the relation between emotion differentiation and empathic accuracy. *Soc. Psychol. Personal. Sci.* 7 240–247. 10.1177/1948550616633504

[B12] Feldman L. A. (1995). Valence focus and arousal focus: individual differences in the structure of affective experience. *J. Personal. Soc. Psychol.* 69 153–166. 10.1037/0022-3514.69.1.153

[B13] Fredrickson B. L. (2001). The role of positive emotions in positive psychology: the broaden–and–build theory of positive emotions. *Am. Psychol.* 56:218. 10.1037/0003-066x.56.3.218 11315248 PMC3122271

[B14] Fredrickson B. L. (2003). The value of positive emotions: the emerging science of positive psychology is coming to understand why it’s good to feel good. *Am. Sci.* 91 330–335.

[B15] Fredrickson B. L. (2004). The broaden–and–build theory of positive emotions. Philosophical Transactions of the Royal Society of London. *Ser. B Biol. Sci.* 359 1367–1377. 10.1098/rstb.2004.1512 15347528 PMC1693418

[B16] Fredrickson B. L. (2013). Positive emotions broaden and build. *Adv. Exp. Soc. Psychol*. 47 1–53. 10.1016/B978-0-12-407236-7.00001-2

[B17] Israelashvili J. Oosterwijk S. Sauter D. Fischer A. (2019). Knowing me, knowing you: emotion differentiation in oneself is associated with recognition of others emotions. *Cogn. Emot.* 33 1461–1471. 10.1080/02699931.2019.1577221 30734635

[B18] Jack R. E. Blais C. Scheepers C. Schyns P. G. Caldara R. (2009). Cultural confusions show that facial expressions are not universal. *Curr. Biol.* 19 1543–1548. 10.1016/j.cub.2009.07.051 19682907

[B19] Jack R. E. Garrod G. B. O. Yu H. Caldara R. Schyns P. G. (2012). Facial expressions of emotion are not culturally universal. *PNAS* 19 7241–7244. 10.1073/pnas.1200155109 PMC3358835 22509011

[B20] Kalokerinos E. K. Erbas Y. Ceulemans E. Kuppens P. (2019). Differentiate to regulate: Low negative emotion differentiation is associated with ineffective use but not selection of emotion-regulation strategies. *Psychol. Sci.* 30 863–879. 10.1177/0956797619838763 30990768

[B21] Kashdan T. B. Barrett L. F. McKnight P. E. (2015). Unpacking emotion differentiation: transforming unpleasant experience by perceiving distinctions in negativity. *Curr. Direct. Psychol. Sci.* 24 10–16. 10.1177/0963721414550708

[B22] Kirby L. D. Tugade M. M. Morrow J. Ahren A. H. Smith C. A. (2014). “Vive la différence: the ability to differentiate positive emotional experience and well-being,” in *Handbook of Positive Emotions*, eds Tugade M. M. Shiota M. N. Kirby L. D. (New York, NY: The Guilford Press), 241–255.

[B23] Lee J. Y. Lindquist K. A. Nam C. S. (2017). Emotional granularity effects on event-related brain potentials during affective picture processing. *Front. Human Neurosci.* 11:133. 10.3389/fnhum.2017.00133 28392761 PMC5364149

[B24] Leventhal A. M. Martin R. L. Seals R. W. Tapia E. Rehm L. P. (2007). Investigating the dynamics of affect: psychological mechanisms of affective habituation to pleasurable stimuli. *Motiv. Emot.* 31 145–157. 10.1007/s11031-007-9059-8

[B25] Lim N. (2016). Cultural differences in emotion: differences in emotional arousal level between the East and the West. *Integr. Med. Res.* 5, 105–109. 28462104 10.1016/j.imr.2016.03.004 PMC5381435

[B26] Lindquist K. A. Barrett L. F. (2008). “Emotional complexity,” in *Handbook of Emotions*, eds Lewis M. Haviland-Jones J. M. Barrett L. F. (New York, NY: The Guilford Press), 513–530.

[B27] Lu L. Gilmour R. (2004). Culture and conceptions of happiness: individual oriented and social oriented SWB. *J. Happ. Stud.* 5 269–291. 10.1007/s10902-004-8789-5

[B28] Lyubomirsky S. (2011). *Hedonic adaptation to positive and negative experiences.* Oxford: Oxford University Press.

[B29] Munir M. Azam R. (2017). Emotional intelligence and employee performance: an intervention based experimental study. *J. Business Econ.* 9 1–19.

[B30] Nelis D. Quoidbach J. Mikolajczak M. Hansenne M. (2009). Increasing emotional intelligence: (How) is it possible? *Personal. Indiv. Diff.* 47 36–41. 10.1016/j.paid.2009.01.046

[B31] Powell A. (2020). *Soothing advice for mad America.* Cambridge: The Harvard Gazette.

[B32] Quoidbach J. Gruber J. Mikolajczak M. Kogan A. Kotsou I. Norton M. I. (2014). Emodiversity and the emotional ecosystem. *J. Exp. Psychol. Gener.* 143:2057. 10.1037/a0038025 25285428

[B33] Sauter D. A. (2017). The nonverbal communication of positive emotions: an emotion family approach. *Emot. Rev.* 9 222–234. 10.1177/1754073916667236 28804510 PMC5542129

[B34] Sauter D. A. Eisner F. Ekman P. Scott S. K. (2010). Cross-cultural recognition of basic emotions through nonverbal emotional vocalizations. *PNAS* 6 2408–2412. 10.1073/pnas.0908239106 PMC2823868 20133790

[B35] Schwarz N. (2012). “Feelings-as-information theory,” in *Handbook of theories of social psychology*, eds Van Lange P. A. M. Kruglanski A. W. Higgins E. T. (New York, NY: Sage Publications Ltd), 289–308. 10.4135/9781446249215.n15

[B36] Selby E. A. Wonderlich S. A. Crosby R. D. Engel S. G. Panza E. Mitchell J. E. (2014). Nothing tastes as good as thin feels: Low positive emotion differentiation and weight-loss activities in anorexia nervosa. *Clin. Psychol. Sci.* 2 514–531. 10.1177/2167702613512794 34888124 PMC8654035

[B37] Shiota M. N. Neufeld S. L. Danvers A. F. Sng O. Yee C. I. (2014). Positive emotion differentiation: a functional approach. *Soc. Personal. Psychol. Comp.* 8 104–117. 10.1111/spc3.12092

[B38] Soscia I. (2007). Gratitude, delight, or guilt: The role of consumers’ emotions in predicting postconsumption behaviors. *Psychol. Market.* 24 871–894. 10.1002/mar.20188

[B39] Tugade M. M. Fredrickson B. L. Feldman Barrett L. (2004). Psychological resilience and positive emotional granularity: Examining the benefits of positive emotions on coping and health. *J. Personal.* 72 1161–1190. 10.1111/j.1467-6494.2004.00294.x 15509280 PMC1201429

[B40] Van der Gucht K. Dejonckheere E. Erbas Y. Takano K. Vandemoortele M. Maex E. (2019). An experience sampling study examining the potential impact of a mindfulness-based intervention on emotion differentiation. *Emotion* 19 123–131. 10.1037/emo0000406 29578747

[B41] Zhou P. Critchley H. Garfinkel S. Gao Y. (2021). The conceptualization of emotions across cultures: A model based on interoceptive neuroscience. *Neurosci. Biobehav. Rev.* 125 314–327. 10.1016/j.neubiorev.2021.02.023 33631316

